# How Digital Therapeutics Are Urging the Need for a Paradigm Shift: From Evidence-Based Health Care to Evidence-Based Well-being

**DOI:** 10.2196/39323

**Published:** 2022-10-20

**Authors:** Merlijn Smits, Geke D S Ludden, Peter-Paul Verbeek, Harry van Goor

**Affiliations:** 1 Department of Surgery Radboud university medical center Nijmegen Netherlands; 2 Faculty of Engineering Technology University of Twente Enschede Netherlands; 3 Faculty of Behavioural, Management and Social Sciences University of Twente Enschede Netherlands

**Keywords:** digital therapeutics, digital health, paradigm shift, paradigm, health policy, health care, evidence, evidence-based, decision-making, challenges, implementation, well-being, digital, technology

## Abstract

A scientific paradigm consists of a set of shared rules, beliefs, values, methods, and instruments for addressing scientific problems. Currently, health care embraces the paradigm of evidence-based health care (EBH). This paradigm prompts health care institutions to base decisions on the best available evidence, which is commonly generated in large-scale randomized controlled trials. We illustrate the application of EBH via the evaluation of drugs. We show how EBH is challenged when it is applied to the evaluation of digital therapeutics, which refers to technology and data to prevent, manage, or treat a medical disorder or disease. We conclude that amid the growing application of digital therapeutics, the paradigm of EBH is challenged in four domains: population, intervention, comparison, outcome. In the second part of this viewpoint, we argue for a paradigm shift in health care so we can optimally evaluate and implement digital therapeutics, and we sketch out the contours of this novel paradigm. We address the need for considering design in health care and evaluation processes, studying user values so that health care can move from a focus on health to well-being, focusing on individual experiences rather than the average, addressing the need for evaluation in authentic use contexts, and stressing the need for continuous evaluation of the dynamic relations between users, context, and digital therapeutics. We conclude that the transition from EBH toward evidence-based well-being would improve the successful implementation of digital technologies in health care.

## Introduction

Digital health refers to all “technology and data that inform medical practice and improve health” [1]. In recent years, investments in digital health have soared. In 2019, the global digital health market was already worth about US $175 billion, and it is expected to reach US $660 billion in 2025 [2]. Such investments are justified by the promises of digital health to improve the quality and efficiency of health care, increase health care accessibility via remote care delivery, and democratize health care for large populations [3]. Also, the World Health Organization (WHO) believes that digital health will help achieve the 17 Sustainable Development Goals [4]. However, given the promising benefits of digital health, it is remarkable that, currently, only a minority of digital health technologies are implemented successfully in health care. Indeed, some authors claim that as many as 98% of all digital health start-ups fail [5]. Not meeting the needs and values of users is identified as one of the major reasons for the unsuccessful implementation of digital health [6].

Digital health can be classified into various categories. Following a recent categorization by the US Food and Drug Administration and the nonprofit association Digital Therapeutics Alliance, in this paper we particularly address the category “digital therapeutics” [7,8]. Digital therapeutics refers to all digital health interventions that are employed to *prevent, manage, or treat a medical disorder or disease* [7,8]. Many digital therapeutics are employed today. Examples are mobile apps for health tracking, medication adherence, or monitoring of blood glucose values [9-11]. In addition, virtual reality (VR) and other forms of serious gaming can be considered digital therapeutics when applied as a means for, among other things, pain distraction, pain therapy, or rehabilitation [4,12,13]. Another important area of digital therapeutics relates to artificial intelligence models to assess, for example, abnormal patient behavior, malignant melanoma, or wounds [14-16]. In all cases, patients or health care providers are interacting with *evidence-based* digital technologies to improve patients’ health [8].

We explain in this viewpoint why the current health care landscape, which we will call the “evidence-based health care paradigm,” does not allow for digital therapeutics to meet user needs and values and, consequently, does not reach successful implementation. Building on our experiences as researchers in digital health, we call for a paradigm shift in health care and sketch out a future paradigm that would enable more successful evaluation and, consequently, implementation of digital therapeutics.

## The Current Paradigm in Health Care: Evidence-Based Health Care

### Paradigm

The theory of *paradigms* by Thomas Kuhn provides a framework for understanding the context in which digital therapeutics are implemented today [17]. In the 1960s, Kuhn published his book, The Structure of Scientific Revolutions, in which he argued that science is not continuously progressing but consists of a set of alternating periods of “normal” science when scientists adhere to a shared set of rules and values. They share what problems are worthy of investigation and what instruments and methods are appropriate for solving problems. Kuhn termed this a paradigm [18]. At a certain point in time, periods of normal science become challenged by anomalies: new phenomena, ideas, or novel methodologies that are incompatible with the current paradigm. This can result in a crisis, which is only solved when a novel paradigm is found that can accommodate such anomalies. A scientific revolution occurs when this novel paradigm is adopted—a process called a paradigm shift [17,18]. We will build on Kuhn’s theory to illustrate why health care is in a digital health crisis and emphasize the need for a paradigm shift to solve the crisis.

Implementation of digital therapeutics generally takes place in the broadly adopted paradigm of evidence-based health care (EBH) or, as it is also termed, evidence-based medicine and evidence-based practice. EBH was developed around 1980 in response to the poor quality of care and high health care costs in the United States [19]. Before its establishment, decision-making was broadly based on expert experience and judgment. EBH aimed to increase the safety, cost-effectiveness, and efficacy of health care while creating an accurate and reliable system for decision-making based on evidence. A typical EBH design and evaluation process was established that particularly guided the development and evaluation of drugs. The process consists of five phases. During phase 1—discovery and development—a new drug is developed in the laboratory. The safety of the drug is tested in phase 2 during preclinical research in laboratory settings. In phase 3, the drug is tested for efficacy via clinical research in people. The drug is reviewed for market approval in phase 4. Phase 5 relates to postmarket safety monitoring of the drug [20]. The collection of evidence is key to these phases of design and evaluation. Evidence is collected, analyzed, and used to inform implementation decisions [21]. Evidence is collected via scientific studies. All studies follow a hierarchy of evidence levels, with the highest-quality evidence created via the systematic review of randomized controlled trials (RCTs). One level lower includes evidence created by RCTs. In an RCT, two or more substantial patient groups are subjected to similar conditions. Only one condition, the intervention, varies. Patients are randomly assigned to one of the groups—ideally blind—to prevent placebo effects. The RCT results in insight into the efficacy of the intervention within the defined patient population. Following the RCT in the evidence tree is evidence from cohort studies, followed by case studies and, finally, expert opinions [22]. The latter three forms of scientific research are rarely considered in health care. Ideally, all evidence in EBH would result from RCTs and their systematic reviews. To guarantee that an RCT generates trustworthy results, studies are standardized by the PICO (population, intervention, comparison, outcome) model [23]. This model prescribes defining the patient or population, intervention, control group, and outcome at the onset of the study.

Today, the need for evaluation results in the tendency to apply the whole EBH pathway—from development and early testing to national implementation—to digital therapeutics [24]. Yet, where this pathway might work for the development and evaluation of drugs, it is not optimal for digital therapeutics. This has been acknowledged before by several authors who provided suggestions on how to evaluate digital therapeutics as part of EBH. Guidelines, for example, exist on the type of research questions to address [25] and the methods of reporting RCTs of digital therapeutics [26]. Also, the WHO has suggested studying acceptability, feasibility, resource use, and gender, equity, and human rights in addition to clinical effectiveness [27]. Nonetheless, even with these guidelines, the gold standard of EBH cannot provide the desired outcomes for achieving a successful evaluation of digital therapeutics resulting in technology meeting the needs and values of all its users. Based on the traditional PICO model of RCTs, we explain below the differences between traditional drug research and digital therapeutics research, and we continue to show why clinical research into the added value of digital therapeutics requires a novel paradigm. Thereby, we do not argue that digital therapeutics do not require evaluation—a view that is termed as “digital exceptionalism” [28]—but we argue that other methods of evaluation are required to understand the added value of technology. For simplicity, we present our arguments as if traditional drug research and research into digital therapeutics are poles. In reality, traditional health is also challenging the strict application of EBH, but this is beyond the scope of this viewpoint [22,29,30].

### Population

The first element of the PICO model traditionally refers to “population” or “patient.” A specific group of patients is identified and studied to evaluate the effectiveness of an intervention. In the evaluation of drugs, the patient population is delimited by a specific medical condition and a specific age group. In digital therapeutics, however, it is generally more difficult to identify one group of patients. Digital therapeutics, for example, might be developed for one medical condition in many age groups (eg, serious gaming for rehabilitation) or for many medical conditions in only one age group (eg, a VR playground for children). It could apply to the entire population (eg, a personal online health tracking app), and it extends beyond disease only (eg, wellness apps focused on the prevention of disease).

Even more challenging than identifying the right patient population is engaging patients in the study. Evaluating digital therapeutics typically involves a time- and effort-intense interaction with a technology that drug research does not require. The health condition of patients could prohibit them from spending time and effort on this interaction, which complicates research [31,32]. As a consequence, today, the involvement of patients in digital therapeutic evaluation often seems to be tokenistic. Typically, only a few users are involved, generating the appearance of diversity and inclusiveness, while many users are left out of the study’s scope or are out of reach [33]. The result? A misalignment between design and the needs and values of all users [6]. To conclude, digital therapeutics challenge the “P” in the PICO model.

### Intervention

We again consider the “intervention” in traditional health care to refer to drug development and its preclinical and clinical evaluation. Phase 1 of drug development starts in the laboratory and follows the rules of EBH. Once a drug is developed, a phase of preclinical evaluation starts, in which the safety effects of the drug are tested in vitro (ie, research in cells) and in vivo (ie, research in animals). The process continues with a clinical evaluation, in which the effects of the drug on the human body are evaluated [20]. A different process is required for digital therapeutics. A digital therapeutic is developed by a team of designers and engineers that conform to different rules, methods, and procedures than those that are known in the EBH paradigm. Design processes, for example, are less structured, rely on qualitative input, and generally do not require evidence for decision-making [24,34]. In addition, designers cannot conduct in vitro evaluation tests but rather they must involve humans directly. Phase 2 of the development pathway is thereby in its current form impossible to conduct. Multiple phases of design and evaluation with users are necessary to design a product that meets user needs. This challenges the current way of evaluating interventions. Consider the example of a VR treatment for chronic pain [35]. First, the current EBH paradigm does not allow for an iterative development and evaluation cycle, while the VR treatment would benefit from cocreation with users. Evaluation with users leads to novel design insights to improve the design, after which a new evaluation cycle should follow [28]. The three typical linear phases of EBH—development, preclinical research, and clinical research—do not support an efficient digital therapeutics design and evaluation process. Second, a typical evaluation study might take months or even years to conduct, with an occasional exception, such as the fast development of vaccines during the COVID-19 pandemic. Digital therapeutics (ie, VR technology) evolves at such a fast pace that an EBH evaluation process only delays progress. With the fast advancements in VR technology, evaluation outcomes might already be outdated once the study ends [24]. Various alternatives have been proposed to RCTs that better meet the needs of digital therapeutic development. For example, a multiphase optimization strategy applied to the RCT allows for adapting of the design during the evaluation process [36]. Also, methods exist to evaluate the principle of a solution rather than the specific technology itself, solving issues of rapid technology advancement [37]. Unfortunately, these novel methods have not been adopted widely [38,39]. To conclude, traditional design and evaluation procedures of EBH do not align with a digital therapeutic as an intervention.

### Comparison

A control group is typically identified in phase 3 of drug research. A comparison of the average results of a large control group with the average results of a large intervention group should show an intervention’s relative effectiveness. Ideally, group allocation is blind to prevent placebo effects. A digital therapeutic questions such an approach on three of its core features: identification of a control group, the placebo effect, and the mean.

First, the creation of a reliable control group in digital therapeutics is challenging [40]. Consider, for example, children with cerebral palsy using a therapeutic digital game for training fine motor skills. These children can be compared with children not using the digital therapeutic solution. These nonusers could include children not receiving any training, although that is generally considered unethical. Nonusers could also refer to children receiving standard training or children receiving the digital intervention in a nondigital way. Related to the example, this would mean that children receive similar exercises that are then visualized in real life, which would make for a poor comparison. Further, a possible control group could include children receiving the training via television, a lower-tech solution. Finally, an interesting control group includes children using a sham placebo to which children using the digital game are compared; the control group children would be using the same digital game without the therapeutic effect and educational lessons in it [41]. Unfortunately, the development of such a sham placebo is expensive. The study outcomes depend on what type of control group is considered, which could challenge the validity of the study.

Second, dealing with placebo effects differs in digital therapeutics. A placebo effect refers to the positive effect of an intervention on a person’s health, not because the intervention has an objective biological effect but because of the subjective psychological effect of a patient believing in the intervention [42]. In the third phase of drug research, the placebo effect is commonly eliminated through blind group allocation to distinguish the objective from the subjective outcomes [43]. Consequently, during implementation in phase 5, clinicians add the placebo effect to the objective biological effect for an optimal therapeutic outcome. In digital therapeutics research, blind group allocation is difficult when not making use of sham interventions. In addition, the distinction between objective outcomes and subjective placebo experiences is difficult to make. Digital therapeutics typically aim at subjective outcomes, such as self-management [44], which challenges the ability to make a distinction between the real effect of a digital therapeutic and its placebo effect [45]. This ultimately challenges the prescription of digital therapeutics, and no consensus exists on the ethical acceptability of prescribing interventions solely based on their placebo effect [46]. Digital therapeutics could, therefore, provide a novel perspective to this ethical debate by challenging the traditional role of placebo in health care.

Finally, the comparison of the mean of two groups is critical in digital therapeutics. Whereas such comparison provides insight into the efficacy of a certain drug, it will not generate the detailed insights that a digital therapeutic requires. In digital therapeutics, the outliers and experiences matter. Consider two patients: patient 1 benefits from using VR for chronic pain whereas patient 2 does not, as this patient needs more technical support for optimal use. The average is a mediocre outcome. The conclusion? The VR treatment is not proven efficacious. It worked for patient 1, and it might have worked for patient 2 once this patient had received additional support. Many individual preferences affect the use of digital therapeutics (eg, the reasons for use, the “dosing of use” [ie, one person might benefit from intense use whereas another desires sporadic use], and the necessary support in use). This requires a move from general outcomes to individual experiences. Promising alternatives to the RCT already exist but are not commonly used. One example is the single-case experimental design, which prescribes studying individual experiences over a longer period while manipulating the treatment [47]. To conclude, the traditional way of using a control group to evaluate an intervention’s effectiveness does not align with the practical reality of evaluating digital therapeutics.

### Outcome

EBH mostly considers the so-called “hard impacts” of a studied intervention as major evidence. Hard impacts are quantitative outcome measures [48]. Examples of outcomes in drug research include the ability to cure disease and cost-effectiveness. These outcomes are identified at the design phase of the study. Improvement of these outcomes justifies implementation. Although a focus on hard impacts was needed to improve the quality of health care two decades ago [49], solely considering hard impacts in digital therapeutics results in missing important insights required to implement them successfully. EBH has been criticized for its overemphasis on cost-effective decision-making [50,51]. So-called “soft impacts” [48], such as social, ethical, and psychological outcomes, are rarely considered. These are particularly important in the context of digital therapeutics as they provide valuable information on the alignment of a design with users’ needs and values [24]. Authors have already stressed the importance of considering these soft impacts in the evaluation of digital therapeutics. Michie et al [52], for example, addressed the need for considering the ethics of digital therapeutics. Maramba et al [53] called for the application of qualitative methods in the evaluation of digital therapeutics. The WHO has recently addressed the need to study user behavior, knowledge, attitude, acceptability, and feasibility [27,54]. Also, concepts such as patient-reported outcome measures and patient-reported experience measures have been introduced to health care [55]. Nonetheless, research practice and reimbursement of digital therapeutics continue to value hard outcomes over soft ones [38,39]. The current paradigm does not motivate studying soft impacts, as these are considered to be low-quality sources of evidence [22]. In addition, these soft impacts cannot always be identified before the study, which challenges the traditional way of evaluating in EBH. Solely focusing on hard outcomes results in the unsuccessful implementation and reimbursement of many valuable digital therapeutics.

In addition to missing important insights required for the successful implementation of digital therapeutics, the narrow focus on hard outcomes in health care prevents the definition of “health” from moving beyond the “absence of disease or infirmity” [56,57]. This definition is also referred to as “negative health.” Several initiatives have aimed to redefine health within the domain of health care toward “positive health” that considers it as “well-being” and aspires to individual flourishing [58-61]. Adoption of positive health remains low [62], but it would do more justice to the opportunities of digital therapeutics to encourage self-management and a healthy lifestyle. A larger focus on soft outcomes in digital therapeutics would, therefore, not only improve the implementation of digital technologies but would also enable health care to move its focus from health to well-being [63].

### Context

A digital therapeutic is not a drug that can be administered with a prescription. It needs support structures and logistics and it requires education and behavior change in patients, care professionals, and other actors involved. For example, the real effect of VR on chronic pain can only be measured reliably when VR is implemented as part of a pain treatment offered by a medical doctor, supported by logistical and technical structures, and properly used by patients. Namely, it is not only the VR technology but the whole health care service supporting it that should lead to effective pain treatment. This way, a digital therapeutic can be seen as a social intervention. All actors, existing health care procedures, and the time of use should be closely addressed for successful implementation. We, therefore, introduce “context” as a novel element of the PICO model (PICCO). Numerous authors have underlined the importance of considering the context of digital therapeutics. Shaw et al [64], for example, addressed the importance of studying the health care team and its current routines. Lehoux and Blume [65] illustrated the importance of considering the sociopolitical context of digital health by identifying all people involved with the digital health solution, the power dynamics between people, the resources necessary to implement digital health, and the knowledge necessary to use it. Likewise, Reuzel et al [66] called for a study of the social context of technology from a “social shaping” perspective for understanding how technology affects the norms and values of the various users. With the importance of context, there is also a need for another order of development phases. Instead of evaluation preceding implementation, digital therapeutics require implementation to precede final evaluation [67]. Hence, the added value of a digital therapeutic can only be reliably measured when the technology is implemented and has become part of standard care. This is problematic as implementation decisions in EBH are currently made based on evaluation outcomes. Despite all frequent requests, spatial and temporal complexities of digital therapeutics are rarely addressed, as the PICO model currently does not allow for consideration of this context [6,34,68].

## A Novel Paradigm for Digital Therapeutics: Evidence-Based Well-being

### From Health to Well-being

Digital therapeutics are creating anomalies in the paradigm of EBH. The PICCO formulation above clearly indicates what anomalies occur when applying EBH to digital therapeutics. A novel transdisciplinary paradigm is required that enables studying the added value of digital therapeutics in health care. Below, we attempt to outline five elements to which a novel digital therapeutics paradigm should adhere. We explain that the full potential of digital therapeutics is only reached when a transition is made from *health* (ie, the absence of disease or infirmity) to *well-being* (ie, a state of persons that designates that they are happy or flourishing and that their life is going well for them) [56,57,69]. We, therefore, name the paradigm “evidence-based well-being.” Rather than disregarding clinical research entirely in health care, this novel paradigm focuses on user experiences of well-being as reliable sources of best available evidence for designing, evaluating, and implementing digital therapeutics in addition to the more objective evaluations of better health, security, safety, and cost-effectiveness.

### Consider Design in Evaluation

Digital therapeutics introduce a novel discipline into the domain of health care: design. The framework of evidence-based design was established to bridge gaps between health care and design by embedding scientific evaluation in design processes [70]. This framework considers an interdisciplinary approach to health care design by adopting principles of EBH in design [71]. Instead, we aim for a transdisciplinary approach in which design and health care form a novel paradigm without forcing one culture onto the other. This requires moving away from the linear processes of design preceding evaluation [72]. Instead, an iterative process should be established in which health care practitioners and designers constantly collaborate and set up multiple design and evaluation phases. Today, health care generally questions the “yes” or “no” regarding the implementation of digital therapeutics, but a more effective collaboration would explore *how* digital therapeutics can optimally benefit health care [36,37,73].

### Consider Values

Digital therapeutics provide many opportunities to positively affect the well-being of patients. Technology can, for example, enable patients to control their health, improve their social relations, and facilitate participation in daily life [35]. Soft outcomes should receive more appreciation to encourage health care to look beyond health and toward well-being. To encourage the adoption of soft outcomes as a source of reliable evidence, evaluation could focus on measuring “values.” These relate to everything that people consider important in life and can include both moral and nonmoral values [74]. But this focus on values should not be confused with the increasingly popular health care delivery model of value-based health care (VBH), which aims to measure health outcomes against the costs of health care delivery [75]. Whereas VBH thereby mainly considers economic value, we call for an improvement in individual values, such as autonomy, safety, and privacy. A values-based focus supports the inclusion of a wide variety of soft, delimited outcomes without needing to identify these before the onset of the study. Multiple tools already exist to design and evaluate for values in digital health, which facilitates the adoption of this viewpoint [76,77]. By adopting a values-based focus, health care could shift from health to well-being, and digital therapeutics can reach their full potential.

### Consider Individual Experiences

Digital therapeutics require a different evaluation methodology than solely considering the RCTs valued by EBH. There is a need for a move beyond the average result of a large group toward an evaluation of a wide variety of individual experiences, while preventing cherry-picking [47]. Obtaining insight into individual experiences enables the personalization of digital therapeutics (eg, its user interface or user experience design and service implementation), which is a key factor in improving adherence and engagement [78]. Not only should health care researchers value such individual experiences and personalized technology, but health care insurance and investors should also share the value of experience to optimally support and implement digital therapeutics.

### Consider the Authentic Context of Use

Digital therapeutics only work when they are part of a supporting health care service. As a result, a design process should not be restricted to the technology. Instead, it should consider designing the complete service that the technology is part of. This includes designing, among other things, interactions between patients and health care professionals, communication lines for expectation management of patients, digital therapeutics distribution lines, and technical support lines. During evaluation, the full service needs to be assessed and optimized. Design and evaluation, therefore, should take place in the authentic context of use. The added benefit of studying technology in the authentic use context is that it facilitates user involvement. User involvement requires adjusting the research tool toward the abilities and time of the various users [79]. Observation of users’ lived experiences (ie, empirical understanding of action and perception in daily context) is an accessible way to involve users in the process [80,81]. Facilitating users to make use of digital therapeutics in their daily lives enables them to spend time on the interaction, without being burdened too much by the research objectives. Such observations provide insight into users’ preferences for digital therapeutics (eg, the ideal time and location of use, frequency of use, and support in use) and enable users to provide recommendations for the design of the solution along the way.

### Consider Dynamism

Once a technology is implemented, it might restructure current practices and relations. Digital therapeutics could, for example, affect how patients experience their health, the workload of care providers, and the relationship between care providers and patients [82,83]. Furthermore, what users considered to be important might change once digital technologies are introduced (ie, value mediation) [84]. A digital therapeutic forms a dynamic web of temporal and spatial relations and interactions. The configuration of the web dictates what function the technology fulfills. The same technology, for example, can be used for prevention, monitoring, and recovery, depending on what support services are established [85]. This requires a different mindset for evaluation. It requires a study into the optimal configuration of the web and a structural re-evaluation and reordering once conditions change, long after initial implementation [86].

## Stimulating a Paradigm Shift

We have argued that the shift from EBH to evidence-based well-being would benefit the design, evaluation, and implementation of digital therapeutics. Yet, what can be done to stimulate a paradigm shift to this novel paradigm? An interesting view on stimulating change is the approach of transition management [87]. The authors of this approach illustrate how changes in complex systems, such as the current EBH paradigm, can be accelerated. They stress the importance of niche creation, frontrunners, and diversity. Based on their recommendations, we propose the following for stimulating a transition in health care:

1. Allocate resources and attention to the creation of niches of digital therapeutics research and implementation.
2. Give audience to, and be inspired by, visionaries within digital therapeutics.
3. Stimulate novel ideas and approaches to digital therapeutics in health care.
4. Establish physical spaces where designers and health care providers work together on digital therapeutics to enable a transdisciplinary culture in health care innovation.

## Conclusion

Amid the growing application of digital health technologies, it is time for a change. In this viewpoint, we have shown that the application of EBH to the clinical evaluation of digital therapeutics is problematic. The current paradigm of EBH is challenged by the introduction of digital therapeutics. Instead of proposing a digital exceptionalism in which digital therapeutics do not need to meet safety standards and clinical efficacy, we have argued for the need for other sources of evidence to inform the design and evaluation of digital therapeutics prior to implementation. Instead of EBH, we proposed the paradigm of evidence-based well-being. In this paradigm, design and evaluation become transdisciplinary fields, values are important outcome parameters, individual experiences are a major source of evidence, research is conducted in users’ authentic context of use, and the dynamics between users, context, and technology are constantly evaluated. In addition to being valuable for digital health, these recommendations might even inspire a novel approach to traditional health (ie, drug research). The anomalies in our traditional scientific paradigm are clear; it is time for a paradigm shift to evidence-based well-being to optimally align digital therapeutics with the needs and values of each user.

## Abbreviations

EBH: evidence-based health care
PICCO: population, intervention, comparison, context, outcome
PICO: population, intervention, comparison, outcome
RCT: randomized controlled trial
VBH: value-based health care
VR: virtual reality
WHO: World Health Organization
